# Orbital Apex Syndrome Due to Aspergillus flavus Infection in Immunocompetent Patients: A Report of Two Cases

**DOI:** 10.7759/cureus.43508

**Published:** 2023-08-15

**Authors:** Salil Mehta, Kanchan Gupta, Neha Patel Nakshiwala

**Affiliations:** 1 Ophthalmology, Lilavati Hospital, Mumbai, IND; 2 Radiology, Lilavati Hospital, Mumbai, IND; 3 Infectious Disease, Lilavati Hospital, Mumbai, IND

**Keywords:** immunocompetent patients, functional endoscopic sinus surgery (fess), complete ophthalmoplegia, orbital apex syndrome, aspergillus fumigatus, invasive fungal infections

## Abstract

*Aspergillus* species are fungi that are commonly found in soil and decaying vegetation and have the potential to cause an orbital apex syndrome that is marked by ophthalmoplegia or vision loss. We report the clinical and investigational findings and outcomes of two patients with orbital apex syndrome.

The first patient was a 26-year-old female, premorbidly healthy, who presented with a gradually increasing proptosis of the left eye with a reduction in vision. An MRI revealed findings consistent with proptosis, pansinusitis with a soft tissue opacity involving the left orbital apex with optic nerve compression, extending to the cavernous sinus with an associated temporal meningeal enhancement. Following functional endoscopic sinus surgery (FESS), *Aspergillus flavus* was grown in culture, and oral voriconazole was initiated.

The second patient was a 53-year-old male who presented with bilateral reduction of vision and ptosis, proptosis with total ophthalmoplegia (third, fourth, and sixth nerve palsies) of the right eye. An MRI study revealed extensive involvement of the apex of the right orbit, the right cavernous sinus, the medial aspect of the left cavernous sinus, and the pituitary gland. A FESS was done, and the histopathology specimen was suggestive of aspergillosis, and the tissue fungal polymerase chain reaction (PCR) test was positive for *Aspergillus flavus*. He was treated with amphotericin B and oral voriconazole with significant improvement.

Physicians need to have a high index of suspicion for invasive fungal sino-orbital infections, even in immunocompetent patients. The presence of nasal congestion, recurrent sinusitis, facial pain, headache, orbital cellulitis, proptosis, or ophthalmoplegia should prompt early investigations.

## Introduction

*Aspergillus *species are fungi that are commonly found in soil and decaying vegetation and have the potential to cause lung and paranasal sinus disease [1]. Subsequent orbital infection can produce fungal cellulitis or an orbital apex syndrome marked by ophthalmoplegia or vision loss. These patients are at risk of significant visual morbidity, hence, prompt diagnosis and treatment are needed. We report the clinical and investigational findings of two patients with orbital apex syndrome and their outcomes.

## Case presentation

Case one

A 26-year-old female, premorbidly healthy, presented to her general practitioner with recurrent symptoms of a cold, an intermittent headache, and a gradually progressive reduction of vision in the left eye for the last two to three months. She received some symptomatic treatment, in the form of loose tablets, but was not relieved. There was the onset of a gradually increasing proptosis of the left eye, with a further reduction of vision. At this point, she underwent a magnetic resonance imaging (MRI) study, which revealed findings consistent with proptosis, pansinusitis, and a soft tissue opacity involving the left orbital apex with optic nerve compression, extending to the cavernous sinus with an associated temporal meningeal enhancement. She was admitted to a local hospital and received an initial treatment of methylprednisolone (1 gram) for two days. Two days later, she underwent extensive functional endoscopic sinus surgery (FESS), and the resultant material was sent for staining and culture. A potassium hydroxide (KOH) stain revealed fungal elements. Two days later, she developed right upper limb weakness and altered sensorium. She was switched over to dexamethasone (8 mg) intramuscular (IM) once a day. A repeat MRI showed a left temporoparietal infarct with a left internal carotid artery (ICA) occlusion. At this point, oral itraconazole was added. On day four, following the FESS, *Aspergillus flavus* was grown in culture, and the oral itraconazole was changed to oral voriconazole (200 mg) twice daily. A day later, conventional amphotericin B was added, and she was managed conservatively. Following 11 days of admission, she took discharge against medical advice and requested a transfer to our hospital.

On admission, she was conscious, oriented, and followed all commands. Baseline parameters included a pulse rate of 94/min, blood pressure readings of 110/70 mmHg, and a respiratory rate of 18/min. A systemic examination showed normal breath sounds, normal heart sounds, and no abdominal tenderness. There was a hemiparesis on the right side, with muscle power being assessed at 2/5 in both the upper and lower limbs in contrast to 5/5 on the left side. Investigations for the presence of diabetes mellitus and antibodies to human immunodeficiency virus (HIV) infection types 1 and 2 were normal.

**Figure 1:**
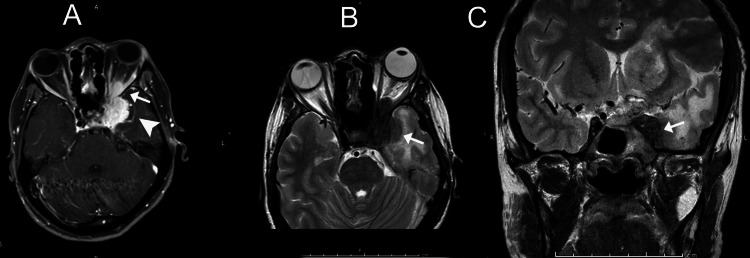
A: An axial T1-weighted image (post-contrast) showing an enhancing soft tissue lesion involving the left cavernous sinus, encasing the left internal carotid artery (ICA) (arrowhead), and extending into the left orbital apex (arrow); B: An axial T2-weighted image showing a hypointense lesion involving the left cavernous sinus (arrow); C: A coronal T2-weighted hypointense lesion involving the left cavernous sinus encasing the left ICA

She was reimaged on an MRI, which revealed an irregular marginated cuff of a soft tissue intensity lesion (T2-weighted (T2)/fluid-attenuated inversion recovery (FLAIR) sequences showed hypointensity; T1-weighted (T1) sequences showed iso-intensity to hyperintensity) measuring 4x2.4x4 cms, involving the left intraconal orbital space extending posteriorly through the optic canal and the superior orbital fissure to involve the temporal lobe and anterior temporal pole, cavernous sinus, and extending inferiorly into the sphenoid sinus and the bilateral sphenopalatine foramen. The mass was completely encasing the distal part of the intraconal portion of the left optic nerve, the optic tract, and the inferior left half of the optic chiasm. It was also causing significant compression of the cavernous and supraclinoid parts of the ICA. The left ICA showed significant luminal narrowing with diffuse circumferential wall thickening with non-opacified left cavernous/supraclinoid middle cerebral artery (MCA) suggestive of thrombosis. On post-contrast views, the lesion showed moderate heterogeneous enhancement with areas of necrosis. Multifocal areas of restricted diffusion with T2/FLAIR hyperintensities were seen in the left fronto-parieto-temporal region, suggesting multi-territorial MCA infarcts, the largest measuring 2x2.4 cm in the left lentiform nucleus, which showed hypointensity within T2/FLAIR. Diffuse heterogenous mucosal thickening was seen in the left maxillary sinus, sphenoid, and frontal sinusitis with moderate post-contrast enhancement. Diffuse leptomeningeal enhancement was also seen along the left parieto-occipital sulci and basal cisterns, with nodular sulcal meningeal-enhancing lesions suggestive of meningitis. The salient features of the MRI are shown in Figures 1A-1C.

A bedside ocular evaluation revealed her visual acuity to be counting fingers at 3 meters in the right eye and hand movements close to the face (HMCF) with the perception of light and accurate projection in the left. There was proptosis and total ophthalmoplegia (third, fourth, and sixth nerve palsies) with ptosis in the left eye. The anterior segment examination was normal bilaterally except for a dilated fixed pupil with no reaction to light in the left eye. Examination of the fundus was normal in all respects for both eyes. A subsequent detailed examination confirmed her visual acuity was 6/9, N6 in the right eye, and HMCF with accurate projection of light in the left eye. A slit lamp examination bilaterally was normal. A clinical diagnosis of left orbital apex syndrome with total ophthalmoplegia was made. Normal sensations on the left side of the face/cheek were normal, suggesting unimpaired trigeminal nerve function.

A baseline systemic investigational panel revealed normal hemoglobin values of 9.70 gm/dl (normal range: 12.0-15.0 gm/dl); total white blood cell (WBC) counts of 8.31 x 10^3 ^cells/μL (normal range: 4.0-10.0 x 10^3^ cells/μL); platelet counts of 233 x 10^3^/mm^3^ (normal range: 7.5-10.5 x 10^3^ /mm^3^); and an elevated C-reactive protein value of 32.60 mg/L (normal range: < 5.0 mg/L).

She was continued on conventional amphotericin B (150 mg) daily intravenously, oral voriconazole (200 mg) twice daily, oral aspirin and atorvastatin combination (75 mg/10 mg), and oral Aldactone (25 mg) twice a day. On day 10, there was a significant reduction in proptosis with an improvement in the visual acuity to counting fingers at 1 meter in the left eye. She was discharged to continue domiciliary treatment but has not subsequently followed up.

Case two

A 53-year-old male, non-diabetic and seronegative for HIV infection, presented with bilateral reduction of vision associated with irrelevant talk, persistent headache, and occasional giddiness for the last two months. Significant past histories included systemic hypertension and cranial surgery. He had undergone a right temporal anterior craniotomy 16 months earlier, and the biopsy result suggested a skull base granulomatous disease. Based on this finding, he had been initiated on four-drug antitubercular therapy. He continued this for five months before switching over to a two-drug regimen. The biopsy specimen was re-examined four months later using periodic acid-Schiff (PAS) and Grocott methenamine silver (GMS) stains on deeper tissue sections, and occasional fungal hyphae were identified. An MRI at this time showed a soft tissue lesion along the anteromedial aspect of the right middle cranial fossa with the development of right-side osteomastoiditis.

On admission, he was systemically stable with a pulse rate of 88/min, a blood pressure of 140/90 mmHg, and a respiratory rate of 18/min. The examination of the respiratory and cardiovascular systems was normal.

An ophthalmic evaluation was performed. The patient denied light perception in either eye. The right eye showed complete ptosis and proptosis with total ophthalmoplegia (third, fourth, and sixth nerve palsies). The left eye had a complete range of motion. Examination of the anterior segment was normal except for bilateral fixed and dilated pupils with no response to direct light stimulation. Fundus evaluation revealed bilateral disc pallor.

Baseline hematological evaluation was essentially normal, with hemoglobin values of 13.4 gm/dl (normal range: 12.0-15.0 gm/dl); a total WBC count of 8.61 x 10^3^ cells/μL (normal range: 4.0-10.0 x 10^3^ cells/μL); platelet counts of 258 x 10^3^/mm^3^ (normal range: 7.5-10.5 x 10^3^/mm^3^); and a serum creatinine count of 0.56 mg/dl (normal range: 0.50-0.90 mg/dl). Serum 1,3, beta (β)-D-glucan and serum galactomannan assays were done, and the results were found to be positive (serum galactomannan was 0.58 ng/ml (normal range: <0.5 ng/ml), serum 1,3, beta-D-glucan was 4.77 pg/ml (normal range: < 0.1 pg/ml), and he was initiated on oral voriconazole.

**Figure 2:**
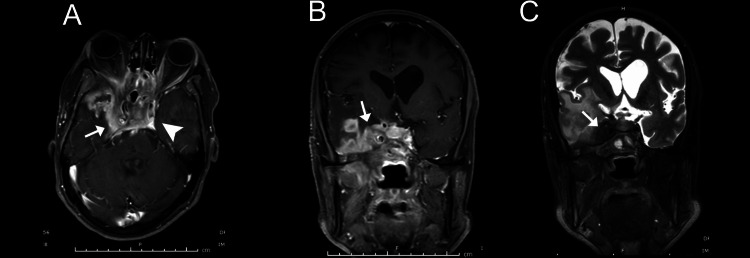
A. An axial T1-weighted image (post-contrast) showing an enhancing soft tissue lesion involving the right cavernous sinus and adjacent temporal region. The left cavernous sinus is normal (arrowhead); B. A coronal T1-weighted image (post-contrast) shows enhancing soft tissue involving the right cavernous sinus and encasing the cavernous segment of the right internal carotid artery (ICA) and adjacent temporal region (arrow); C. A coronal T2-weighted image showing a hypointense lesion involving the right cavernous sinus encasing the right ICA (arrow).

An MRI was repeated, and it revealed a mass measuring 46x58x28 mm involving the anterior and medial aspects of the right temporal lobe with extensive surrounding vasogenic edema. There was extensive involvement of the apex of the right orbit, the right cavernous sinus, the medial aspect of the left cavernous sinus, and the pituitary gland. The salient features of the MRI are shown in Figures 2A-2C.

A lumbar puncture was done, which revealed elevated protein levels (125 mg/dl). Subsequently, functional endoscopic sinus surgery (FESS) was done, and the resultant biopsy material was investigated. The histopathology specimen was suggestive of *Aspergillosis*, and the tissue fungal polymerase chain reaction (PCR) was positive for *Aspergillus flavus*. Other results included GeneXpert testing (negative) and pyrosequencing for drug-resistant tuberculosis (negative).

Additionally, intravenous conventional amphotericin B (50 mg diluted in 500 ml) was added to the oral voriconazole being administered (200 mg) twice daily. He was also administered dexamethasone intravenously (4 mg) three times a day for seven days. After 10 days, he noticed a reduction in proptosis on the right side and a recovery of vision on the left side due to hand movements and accurate projection of light. He was subsequently discharged on oral voriconazole (200 mg) twice daily and advised to follow up.

## Discussion

The fungi of *Aspergillus spp*. are commonly found in the soil and vegetative material, and common species include *Aspergillus fumigatus* complex, followed by *A flavus*, *A niger*, and *A terreus*. *Aspergillus fumigatus* is the commonest pathogen, followed by *Aspergillus flavus*, which is more commonly seen in warmer climates due to its innate ability to survive at higher ambient temperatures [1]. Several mechanisms exist to provide a protective response to the host organism. Initially, airway cilia play a role in the removal of spores. Subsequently, airway alveolar macrophages and recruited neutrophils are important mechanisms for an immune response. The pathogen-host interaction causes a wide spectrum of diseases. A hypersensitivity reaction to inhaled spores can cause allergic aspergillosis, whereas patients with structural lesions of the lung may develop aspergilloma. Invasive and angio-invasive diseases are common in the presence of significant immunosuppression, such as neutropenia, immunosuppressive therapy, diabetes mellitus, or tropical residence. In these cases, hyphae may infiltrate tissue planes and cause hematogenous dissemination [2]. The mortality of invasive aspergillosis is estimated to be 30%-80% [3]. Invasive infections are being increasingly reported in immunocompetent patients.

Invasive fungal infections of the paranasal sinuses are increasingly frequent and may initially be difficult to diagnose. Resident spores, under specific conditions, may become saprophytic and multiply. These invasive masses may produce fungal sinusitis, and the close anatomical proximity to the orbit often permits the spread of infection into the orbit and via the superior orbital fissure to the middle cranial fossa.

Several authors have reported on the clinical features and treatment modalities of this group of patients. Adulkar reported on the clinical features of 20 immunocompetent patients with invasive sino-orbital disease from a center in South India. *Aspergillus spp.* was the pathogenic organism in 18 cases, while mucormycosis was detected in two cases. Common presentations include proptosis (15 patients), diplopia (five patients), and reduced vision (four patients). The average time before presentation was six months, and most patients had received a range of diagnoses varying from idiopathic orbital inflammation to cellulitis. All patients underwent surgical debridement and systemic antifungal therapy (amphotericin B/itraconazole). Additionally, some patients needed a maxillectomy or exenteration [4]. Heier reported four immunocompetent patients with invasive fungal sino-orbital disease who presented with proptosis for durations ranging from four to 72 weeks. Subsequent imaging and sinus biopsy cultures grew *Drechslera spp.* (two patients), *Aspergillus fumigatus* (one patient), and *Curvularia spp.* (one patient). Antifungal therapy (amphotericin B and/or itraconazole) was administered to all patients [5]. In an extensive review, Rupa and co-workers analyzed 147 papers on chronic granulomatous invasive fungal sinusitis. The analysis revealed a preponderance of middle-aged adults from warm tropical countries such as Sudan, India, and Saudi Arabia. Orbital lesions with proptosis (88.2%) were the most common sign, with the ethmoid and maxillary sinuses most involved. *Aspergillus flavus* (64%) was the most common organism reported [6]. Yuan et al., in a comprehensive review of 73 patients with fungal orbital apex syndrome, reported that visual loss and periocular pain were the most common symptoms. Pre-existing risk factors included uncontrolled diabetes mellitus and immunosuppressive treatment. On average, it took 7.4 weeks to diagnose, and 57 of 73 (78%) confirmed *Aspergillus *fungal infection [7].

The commonest differential diagnosis is mucormycosis, which is caused by filamentous molds within the class *Zygomycetes *and order *Mucorales*. An initial clinical diagnosis is complemented by the detection of fungi in nasal or orbital biopsy specimens, either through staining methods or culture. The characteristic appearance of mucormycosis on potassium hydroxide (KOH) mounts, preferably with calcofluor white, generally shows wide, ribbon-like non-septate filaments that branch at right angles. On histopathological examination, specimens show broad, aseptate fungal hyphae with an inflammatory cell infiltrate, often with a necrotic background, often clustered around blood vessels (angioinvasive). In addition, the development of PCR techniques, including nested PCR or real-time PCR (qPCR), may be helpful [8]. The early differentiation of mucormycosis is necessary to allow appropriate therapy with systemic amphotericin B or the newer triazoles such as posaconazole or isavuconazole, in contrast to the voriconazole recommended for *Aspergillus *infection.

Case one had an initial phase of several weeks of non-specific symptoms suggestive of sinusitis, which failed to respond to presumably symptomatic treatment. The onset of proptosis led to a more comprehensive evaluation, including imaging and a sinus biopsy, which led to the diagnosis. In the interim, she received several doses of corticosteroids for a possible diagnosis of optic neuritis or idiopathic orbital inflammation, which may have exacerbated the disease. Similarly, case two had several months of anti-tubercular treatment, based on an earlier histopathology study, before imaging and a sinus debridement surgery revealed the pathogenic organism. This patient also had a positive result for serum galactomannan and 1,3-beta-d-glucan assays. Galactomannan is a polysaccharide that is specific to the cell wall of *Aspergillus*. It is detectable in the serum during invasive infections and may be detectable up to a week before clinical findings. 1,3-beta-d-glucan is a panfungal marker and may be detected in invasive infections due to a wide variety of fungi, including *Aspergillus*, *Candida*, *Fusarium*, and *Trichosporon *[9].

These cases we describe share some similarities with the previous series. These patients also presented with proptosis, ophthalmoplegia, and reduced vision (unilaterally or bilaterally) and were immunocompetent with no apparent risk factors. Both eventually underwent surgical debridement for the purposes of acquiring tissue for histopathological and microbiological examination, thus leading to a diagnosis of *Aspergillus flavus* infection. Both patients significantly improved following a combination of amphotericin B and voriconazole therapy, with a reduction in proptosis and partial visual recovery, but a significant temporal delay preceded the diagnosis.

## Conclusions

We describe the clinical and investigational findings of two immunocompetent patients with orbital apex and cavernous sinus disease due to *Aspergillus flavus* infection. Imaging studies and microbiological analysis led us to the correct diagnosis following a significant delay. While the patients significantly improved following a combination of amphotericin B and voriconazole therapy, a delayed diagnosis could potentially have led to a worse anatomical and visual outcome.

We suggest that physicians need to have a high index of suspicion for invasive fungal sino-orbital infections, even in immunocompetent patients. The presence of nasal congestion, recurrent sinusitis, facial pain, headache, orbital cellulitis, proptosis, or ophthalmoplegia should prompt early imaging studies followed, if needed, by functional endoscopic sinus surgery to debulk as well as permit KOH staining, histopathology, or PCR of the tissue samples.
